# Optimum Design of InGaN Blue Laser Diodes with Indium-Tin-Oxide and Dielectric Cladding Layers

**DOI:** 10.3390/nano14171409

**Published:** 2024-08-28

**Authors:** Chibuzo Onwukaeme, Han-Youl Ryu

**Affiliations:** Department of Physics, Inha University, 100 Inha-ro, Michuhol-gu, Incheon 22212, Republic of Korea; adonwuka@inha.ac.kr

**Keywords:** InGaN, laser diode (LD), indium tin oxide (ITO), wall-plug efficiency (WPE)

## Abstract

The efficiency of current GaN-based blue laser diodes (LDs) is limited by the high resistance of a thick p-AlGaN cladding layer. To reduce the operation voltage of InGaN blue LDs, we investigated optimum LD structures with an indium tin oxide (ITO) partial cladding layer using numerical simulations of LD device characteristics such as laser power, forward voltage, and wall-plug efficiency (WPE). The wall-plug efficiency of the optimized structure with the ITO layer was found to increase by more than 20% relative to the WPE of conventional LD structures. In the optimum design, the thickness of the p-AlGaN layer decreased from 700 to 150 nm, resulting in a significantly reduced operation voltage and, hence, increased WPE. In addition, we have proposed a new type of GaN-based blue LD structure with a dielectric partial cladding layer to further reduce the optical absorption of a lasing mode. The p-cladding layer of the proposed structure consisted of SiO_2_, ITO, and p-AlGaN layers. In the optimized structure, the total thickness of the ITO and p-AlGaN layers was less than 100 nm, leading to significantly improved slope efficiency and operation voltage. The WPE of the optimized structure was increased relatively by 25% compared to the WPE of conventional GaN-based LD structures with a p-AlGaN cladding layer. The investigated LD structures employing the ITO and SiO_2_ cladding layers are expected to significantly enhance the WPE of high-power GaN-based blue LDs.

## 1. Introduction

After the first development of III-nitride laser diodes (LDs) in 1996 [1], the performance of blue LDs has improved considerably. To date, GaN-based blue laser diodes have attracted considerable attention as light sources for laser projection displays [2,3,4], high-luminance laser-based lighting [5,6,7,8], visible light communications [9,10,11], and laser-based materials processing [12,13,14,15]. In particular, due to the higher absorption of blue light in copper material compared to near-infrared radiation, there is an increasing demand for high-power blue LDs for welding and cutting applications of copper-based materials [13,15]. In recent years, the performance of blue LDs has been improved considerably, with laser output power >5 W, threshold current density (*J_th_*) <1 kA/cm^2^, slope efficiency (SE) >2 W/A, and wall-plug efficiency (WPE) >40% [3,4,13,14,15,16,17]. However, the performance of GaN-based blue LDs is still inferior to that of GaN-based blue LEDs or GaAs-based infrared LDs, demonstrating a WPE of more than 70% [18,19,20].

The performance of GaN-based LDs is mainly limited by the high growth temperature and electrical resistivity of p-type AlGaN cladding and electron-blocking layers. The typical growth temperature for InGaN QWs of blue LDs ranges from 700 to 800 °C, while the optimum growth temperature is >1000 °C for the AlGaN p-cladding layer. Since the growth temperature of the AlGaN layers is significantly higher than that of InGaN quantum-well (QW) active layers, severe thermal damage can be caused to the QWs during epitaxial growth of a thick p-AlGaN cladding layer [21,22]. To mitigate this issue, the p-AlGaN is typically grown at a temperature much lower than its optimum growth temperature, resulting in poor crystal quality and high resistivity. Moreover, the high acceptor activation energy of Mg in (Al)GaN and the low mobility of hole carriers considerably increase the resistivity of p-type doped layers. Since the p-type AlGaN cladding layer in an LD structure should be sufficiently thick (>0.5 μm) to confine a lasing mode in the vertical direction, the existence of a low-conductivity p-AlGaN cladding layer is the primary reason for high operation voltage, limiting the WPE of GaN-based LDs [18,23].

One promising approach for reducing the operation voltage of GaN-based LDs is to replace part of the p-AlGaN cladding layer with indium tin oxide (ITO), which is conductive and transparent in the visible wavelengths [24,25,26,27,28,29]. ITO is widely used as a transparent current-spreading layer in industry and can form ohmic contacts to p-GaN with low specific contact resistance [24]. The resistivity of p-type (Al)GaN materials is known to be >1 Ωcm [25]. On the other hand, the resistivity of ITO material was reported to be as low as 10^−4^ Ωcm [30]. The refractive index of ITO is around 2, which provides sufficient optical confinement for the laser mode. Therefore, the use of ITO as a part of the p-type cladding region allows a thinner p-AlGaN cladding layer, which could result in reduced operation voltage. Improved LD performances were reported for GaN-based green LD structures with the ITO cladding layer experimentally [27]. In addition, a reduction in the threshold current density from InGaN green LDs with the ITO cladding was reported by theoretical studies in Refs. [28,29]. On the contrary, performance improvements were not clearly demonstrated in the case of blue LD structures with the ITO cladding layer, compared to the conventional blue LD structures with AlGaN p-cladding layers.

In this work, we investigate the optimum InGaN blue LD structure with the ITO partial cladding layer using numerical simulations. The p-cladding region of the LD structure consists of p-AlGaN and ITO layers, and the thicknesses of the p-AlGaN and ITO layers are optimized to exhibit high WPE. In the optimum structure, the thickness of the p-AlGaN layer can be significantly reduced, which leads to decreased operation voltage and increased WPE. In addition, we propose a new type of LD structure having a thin ITO layer and a dielectric partial cladding layer on p-AlGaN layers. In this structure, the cladding region above the active region consists of p-AlGaN, ITO, and dielectric layers. It will be shown that the thickness of p-AlGaN and ITO layers can be greatly reduced to less than 100 nm, leading to significantly improved efficiency characteristics compared to existing GaN-based blue LD structures. Because of the shortened p-AlGaN cladding layer of LD structures having ITO and dielectric cladding layers, thermal damage in the QW active region could be greatly alleviated during the high-temperature growth of p-AlGaN layers.

For the simulation of this study, we employed the simulation software, laser technology integrated program (LASTIP), produced by Crosslight Software Inc., Burnaby, BC, Canada, https://www.crosslight.com (accessed on 1 August 2023) [31]. LASTIP has been widely used for the numerical investigation of semiconductor laser characteristics. In this work, the thicknesses of p-AlGaN and ITO layers in the LD structures with ITO or SiO_2_ partial cladding layers are optimized to obtain the highest WPE operation.

## 2. LD Structure and Simulation Method

### 2.1. LD Structure

**Figure 1:**
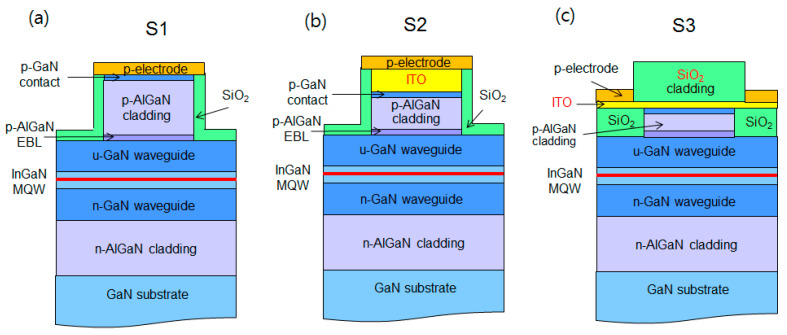
Schematic of the simulated LD structures. (**a**) Conventional LD structure with a p-AlGaN cladding layer (S1); (**b**) LD structure with ITO/p-AlGaN cladding layers (S2); (**c**) Proposed LD structure with SiO_2_/ITO/p-AlGaN cladding layers (S3).

Figure 1 shows the schematic of blue LD structures used for the simulation of this study. The LD structures in Figure 1a–c correspond to the conventional GaN-based blue LD with a p-AlGaN cladding layer (S1), the LD structure with p-AlGaN and ITO cladding layers (S2), and the proposed LD structure with p-AlGaN, ITO, and SiO_2_ cladding layers (S3), respectively. Except for the p-cladding region, the epitaxial layer structures for S1, S2, and S3 are almost identical and similar to those employed in our previous studies [32,33]. The epitaxial layers are composed of a 1.2 μm thick n-type Al_0.04_Ga_0.96_N cladding layer, a 300 nm thick n-type GaN lower waveguide layer, a multiple-quantum-well (MQW) active region, a 200 nm thick GaN upper waveguide layer, a 10 nm thick p-Al_0.2_Ga_0.8_N electron-blocking layer (EBL), a p-type Al_0.05_Ga_0.95_N cladding layer, and a 20 nm thick p-type GaN contact layer. The active region consists of two 3 nm In_0.15_Ga_0.85_N QW layers separated by a 10 nm In_0.02_Ga_0.98_N barrier layer. For this MQW structure, the emission wavelength of the LD was 450 nm at 25 °C. The thicknesses of the lower and upper GaN waveguide layers were chosen to obtain a high optical confinement factor (OCF) and, consequently, a low threshold current could be obtained [32,34]. According to our previous simulation result, the Mg doping concentration of the p-AlGaN EBL and p-AlGaN cladding layers were set to 4 × 10^19^ and 2 × 10^19^ cm^−3^, respectively [30].

In S1, a ridge structure is formed by the dry etching of p-GaN contact and p-AlGaN cladding layers, and the surfaces exposed by the dry etching process are covered with SiO_2_. Then, a p-type electrode is deposited on the p-GaN contact layer. Here, Pd was employed for the p-electrode. On the other hand, in the LD structure S2, ITO is deposited on p-GaN, and both p-Al_0.04_Ga_0.96_N and ITO layers act as p-cladding layers. A ridge shape is formed by the dry etching of ITO, p-GaN contact, and p-AlGaN cladding layers. Then, a p-type electrode is deposited on ITO for current injection. The thicknesses of p-AlGaN cladding and ITO layers in S2 can be reduced compared to the p-AlGaN cladding layer in S1. However, the p-AlGaN and ITO layers in S2 should be thick enough to avoid optical absorption in the p-type metal electrode.

In the case of S3, after growing the relatively thin p-AlGaN cladding and p-GaN contact layers, a ridge structure is formed by dry etching and is insulated by SiO_2_. Then, a thin ITO layer is deposited on p-GaN for the current injection. A SiO_2_ dielectric layer is subsequently deposited on ITO, which serves as the second cladding layer. In S3, since ITO plays a major role in current injection and the mode confinement is achieved by p-AlGaN and SiO_2_ layers, the thickness of ITO can be greatly shortened to less than 50 nm. Therefore, the optical loss associated with ITO is expected to be significantly reduced. A p-electrode is placed on both sides of the SiO_2_ cladding and contacts with ITO for lateral current injection. Since the laser mode does not overlap the p-electrodes and the SiO_2_ layer does not absorb laser light, the modal loss of S3 can be significantly reduced by employing very thin p-AlGaN and ITO layers. The thin p-AlGaN layer is also advantageous for low operation voltages. In S3, optimizing the ITO thickness would be important because there is a tradeoff between resistance and optical loss as the ITO thickness changes.

In all cases of S1, S2, and S3, the residual thickness from the upper boundary of the active region to the bottom of the ridge was set to 200 nm. For the simulations of this study, a ridge width of 10 μm and a cavity length of 1200 μm were chosen. The width of the SiO_2_ cladding layer in S3 was set to 15 μm so that the laser mode did not touch the p-type electrode. The reflectance of the front and rear facets was assumed to be 5% and 98%, respectively. In the simulation, we investigated the laser characteristics by varying the thickness of the p-AlGaN and ITO layers of the three LD structures.

### 2.2. Simulation Method

The LD device characteristics, such as laser output power versus current relation (*L*–*I* curve) and forward voltage versus current relation (*V*–*I* curve), were simulated using LASTIP. This program self-consistently solves the QW band structures, drift and diffusion equation of carriers, radiative and nonradiative carrier recombination, and photon rate equations [31]. The built-in polarization fields were included at the InGaN/GaN, AlGaN/GaN, and InGaN/AlGaN hetero-interfaces using the model reported in Ref. [35], assuming 25% compensation of the polarization fields [36,37,38]. The conduction band offset of the InGaN/GaN and AlGaN/GaN interfaces was set to 0.7 [18]. The incomplete ionization of Mg acceptors in the p-type-doped layers was included such that the acceptor ionization energy in the GaN and AlGaN layers scaled linearly from 170 meV (GaN) to 470 meV (AlN) [18,37], resulting in the acceptor ionization energy of 230 and 180 meV for the p-Al_0.2_Ga_0.8_N EBL and p-Al_0.05_Ga_0.95_N cladding layers, respectively. The mobility model described in Refs. [39,40,41] was used for the mobility of electrons, which resulted in an electron mobility of ~500 cm^2^/Vs for n-GaN with a doping concentration of 1 × 10^18^ cm^−3^. The hole mobilities in the AlGaN, GaN, and InGaN layers were assumed to be 15, 10, and 5 cm^2^/Vs, respectively [31,40].

The refractive indices of the GaN, Al_0.04_GaN, Al_0.05_GaN, and Al_0.2_GaN layers at 450 nm were respectively set to 2.444, 2.422, 2.417, and 2.362, using the refractive index formula and data for AlGaN and InGaN alloys in Refs. [24,42,43]. For the optical absorption loss, we adopted the first-principle calculation model for free-carrier absorption in Ref. [44], which showed an absorption cross-section of ~0.6 × 10^−18^ and ~0.9 × 10^−18^ cm^2^ for n-type and p-type doped layers, respectively [33]. Then, the absorption coefficients of p-AlGaN EBL and p-AlGaN cladding layers correspond to 36 and 18 cm^−1^, respectively. Since the doping concentration of p-type doped layers is almost ten times higher than that of n-type doped layers, the p-AlGaN and p-GaN layers significantly influence the optical loss of GaN-based LDs. The refractive index and absorption coefficient of ITO at 450 nm were respectively set to 2.0 and 2000 cm^−1^, taken from Ref. [24]. For the p-metal electrode, Pd was employed with an absorption coefficient of 890,000 cm^−1^ [45]. The resistivity of ITO was set to 2 × 10^−4^ Ωcm for the simulation of this work.

**Figure 2:**
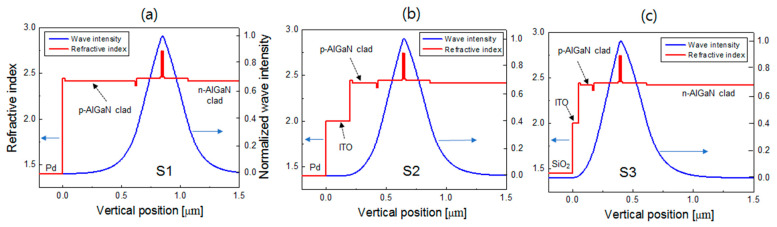
Vertical profiles of the refractive index (left vertical axis) and normalized wave intensity of the lasing mode (right vertical axis) for the LD structure. (**a**) S1 with the p-AlGaN thickness of 600 nm; (**b**) S2 with the p-AlGaN thickness and the ITO thickness of 200 nm; (**c**) S3 with the p-AlGaN thickness of 75 nm and the ITO thickness of 20 nm.

Figure 2 illustrates the profiles of the refractive index and wave intensity of the lasing mode in the vertical direction for S1, S2, and S3. In Figure 2a, the thickness of the p-AlGaN cladding layer is 600 nm. In Figure 2b, the thicknesses of both the p-AlGaN cladding and ITO layers are 200 nm. In Figure 2c, the thicknesses of the p-AlGaN cladding and ITO layers are 75 and 20 nm, respectively. As described later, these thicknesses are close to the optimum values for high WPE operation. S1 and S2 exhibit a similar mode intensity distribution in the vertical direction. For all LD structures, lasing modes are well-confined inside the MQW active region and waveguide layers. The lasing modes are centered near the MQW layers, with an OCF of ~1.5%. In S2 and S3, it can be seen that the lasing mode is effectively confined to a relatively thin p-AlGaN cladding layer owing to the low-refractive-index ITO or SiO_2_ layers. In particular, for S3, the lasing mode is still robustly confined to less than 100 nm total thickness for the p-AlGaN and ITO layers, thereby reducing the operation voltage and optical absorption in p-type layers.

In the carrier recombination model of LASTIP, the radiative recombination rate is calculated by integrating the spontaneous emission spectrum with a Lorentzian line-shape function. The Shockley–Read–Hall (SRH) recombination lifetime was assumed to be 50 ns. However, the effect of SRH recombination on the threshold current was found to be almost negligible when the SRH lifetime was longer than 10 ns. The lasing threshold of InGaN blue LDs is strongly influenced by the Auger recombination coefficient (*C*) [23,46]. For the simulation performed in this work, *C* was chosen to be 2 × 10^30^ cm^−6^/s for the simulated blue LD to exhibit a threshold current density of ~1 kA/cm^2^, similar to those reported recently in high-performance blue LDs [3,13,16,17].

To further confirm the validity of the model and parameters for the simulation of this work, the *L*–*I* curve for the blue LD structure in Ref. [16] was simulated and compared with the experimental data. The LD structure in Ref. [16] employed two 2.5 nm InGaN QWs, 350 nm p-AlGaN cladding, and ITO cladding layers similar to the LD structure S2 in Figure 1b. The ridge width and cavity length were 30 and 1200 μm, respectively. The simulated threshold current and slope efficiency were 0.5 A and 1.9 W/A, respectively. These values are almost identical to those of the experimented LD, confirming that the simulation model and parameters of this study can successfully reproduce the experimental results.

## 3. Results and Discussion

### 3.1. Modal Loss of Simulated Structures

**Figure 3:**
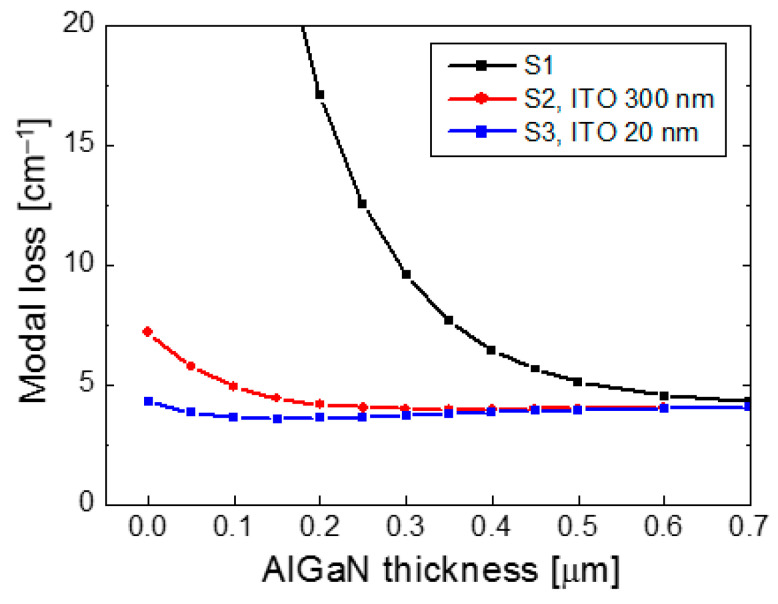
Modal loss of the lasing mode is plotted as a function of the AlGaN thickness for S1, S2, and S3. The ITO thickness of S2 and S3 is 300 and 20 nm, respectively.

Figure 3 shows the modal loss of the lasing mode as a function of the p-AlGaN thickness for S1, S2, and S3. The ITO thicknesses of S2 and S3 were 300 and 20 nm, respectively. The modal loss of S1 decreased rapidly as the AlGaN thickness increased, and it decreased slowly when the AlGaN was thicker than ~500 nm. This implies that the optimum p-AlGaN thickness in S1 could be very thick, though the operation voltage increased with increasing p-AlGaN thickness. The modal loss of S2 and S3 was significantly lower than that of S1, resulting from the strong mode confinement of the low-index ITO and SiO_2_ layer.

In the case of S2, the modal loss was almost invariant to the AlGaN thickness and remained around 4 cm^−1^ when the p-AlGaN was thicker than 200 nm, and it began to increase as the AlGaN thickness decreased from 200 nm. This means that the optimal AlGaN thickness exists around 200 nm, significantly thinner than the optimal AlGaN thickness in S1. For S2, the modal loss was found to be almost independent of the ITO thickness when it was more than 200 nm. When the ITO thickness in S2 was thinner than 150 nm, the modal loss increased significantly as the ITO thickness decreased because the laser mode could pass through a relatively thin ITO layer, and some of the laser light was dissipated from the Pd electrode.

In the case of S3, the modal loss was lower than that of S2 for the AlGaN thickness less than 300 nm due to the significantly reduced ITO thickness of 20 nm. The low modal loss of ~4 cm^−1^ was maintained, even for a thin AlGaN layer of <100 nm, implying that the optimum AlGaN thickness for low optical loss and low voltage in S3 may exist at less than 100 nm, about half of the optimum AlGaN thickness in S2. Therefore, more improved efficiency characteristics are expected from S3.

### 3.2. Device Performance of Conventional LD Structure (S1)

**Figure 4:**
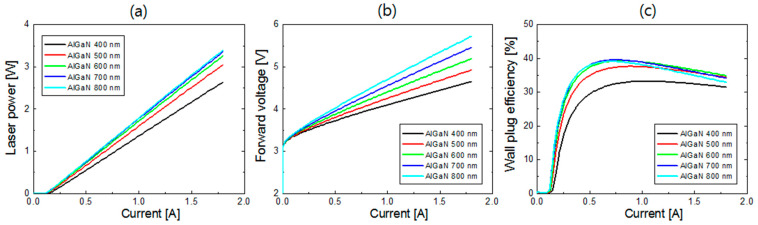
LD device performances of S1 for p-AlGaN thicknesses from 400 to 800 nm. (**a**) Light output power versus current (*L*–*I*) curve; (**b**) forward voltage versus current (*V*–*I*) curve; (**c**) wall-plug efficiency (WPE) as a function of current.

First, the LD device performances of S1 were investigated as the thickness of the p-AlGaN cladding layer varied. Figure 4 shows *L*–*I*, *V*–*I*, and WPE curves for AlGaN thicknesses from 400 to 800 nm. In the *L*–*I* curves, as the AlGaN thickness increased, the threshold current decreased slightly from 0.15 to 0.12 A, and the SE increased from 1.6 to 2.0 W/A. Therefore, laser power at a given current decreased as the AlGaN thickness was reduced, mainly resulting from increased light absorption in the Pd electrode with the decreasing AlGaN thickness. On the other hand, the forward voltage increased significantly as the AlGaN thickness increased, implying that the resistance of the p-AlGaN cladding layer depends strongly on the p-AlGaN thickness.

The WPE curves in Figure 4c were obtained using the *L*–*I* and *V*–*I* results. WPE increased as the AlGaN thickness increased from 400 to 700 nm owing to the decreased optical absorption in Pd, and it began to decline when the AlGaN was thicker than 800 nm, where the voltage effect became dominant. The highest WPE was obtained as 39.8% when the AlGaN thickness was 700 nm. The simulation results on *J_th_*, SE, and WPE for the optimum case are similar to those reported for recently reported high-performance blue LDs [3,13,16,17]. The result of Figure 4 shows the tradeoff between laser power and voltage according to the change in the AlGaN thickness.

### 3.3. Device Performance of ITO/p-AlGaN Cladding Structure (S2)

**Figure 5:**
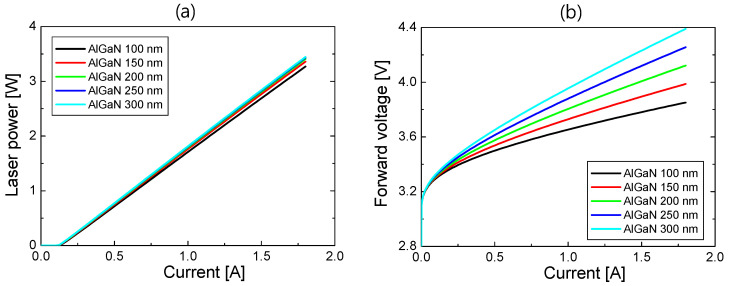
(**a**) *L*–*I* curves and (**b**) *V*–*I* curves of S2 for the p-AlGaN thicknesses from 100 to 300 nm when the ITO thickness is 300 nm.

Next, we investigated the LD device performances of S2 as the thicknesses of the p-AlGaN and ITO layers varied. Figure 5a,b respectively show the *L*–*I* and *V*–*I* curves for AlGaN thicknesses from 100 to 300 nm when the ITO was 200 nm thick. In the *L*–*I* curves, as the AlGaN thickness increased, the threshold current remained at ~0.12 A and SE increased slightly from 1.95 to 2.04 W/A, indicating that the optical loss increased only slightly when the p-AlGaN thickness was reduced. For AlGaN thicknesses between 200 and 300 nm, the *L*–*I* curves look almost identical, which can be understood from the modal loss calculation in Figure 3. In contrast, the *V*–*I* curves are strongly influenced by the p-AlGaN thickness. Like the *V*–*I* curves of S1 shown in Figure 4b, the forward voltage increased with the increasing p-AlGaN thickness due to the increased series resistance of p-AlGaN layers.

**Figure 6:**
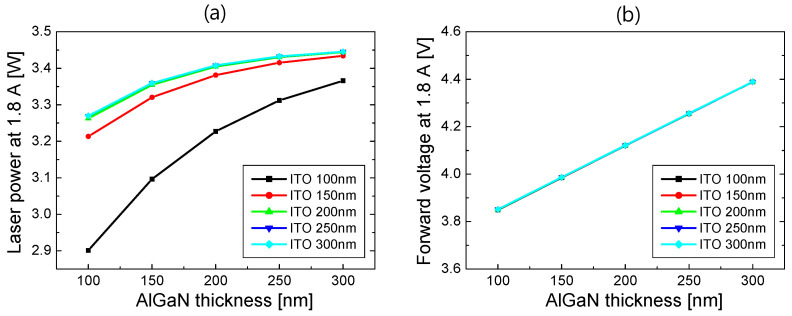
(**a**) Laser power at 1.8 A and (**b**) forward voltage at 1.8 A for the structure S2 are plotted as a function of the AlGaN thickness for ITO thicknesses from 100 to 300 nm.

In Figure 6, laser power and forward voltage at 1.8 A are plotted as a function of the AlGaN thickness for ITO thicknesses from 100 to 300 nm. As the thickness of ITO or AlGaN increased, the optical loss by the Pd electrode decreased, resulting in an increase in SE and laser power. When the ITO thickness was above 200 nm, the laser power was almost invariant to the ITO thickness. This implies that the laser mode was sufficiently confined to AlGaN and ITO layers with little light absorption in Pd. As shown in Figure 6b, the forward voltage increased in proportion to the AlGaN thickness. However, it is almost independent of the ITO thickness, implying that the resistivity of ITO is negligibly small compared to that of the p-AlGaN cladding layer.

**Figure 7:**
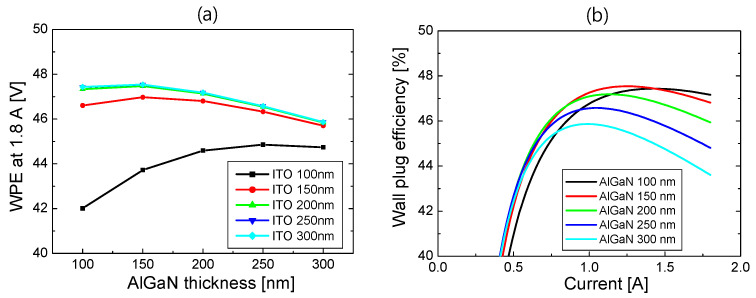
(**a**) WPE at 1.8 A for S2 is plotted as a function of the AlGaN thickness for ITO thicknesses from 100 to 300 nm; (**b**) WPE of S2 as a function of current for the AlGaN thicknesses from 100 to 300 nm when the ITO thickness is 300 nm.

Combining the simulation results of the *L*–*I* and *V*–*I* curves for S2, WPE was calculated as a function of current. In Figure 7a, the WPE at 1.8 A is plotted as a function of the AlGaN thickness for ITO thicknesses from 100 to 300 nm. The WPE increased with the increasing ITO thickness, and it was almost the same for ITO thicknesses above 200 nm, as seen from the results in Figure 5 and Figure 6. When the ITO thickness was more than 150 nm, the highest WPE could be obtained at the AlGaN thickness of 150 nm, where the forward voltage and optical loss are well-balanced. Figure 7b shows WPE as a function of current for the AlGaN thicknesses from 100 to 300 nm when the ITO thickness is 300 nm. The WPE exceeded 45% when the injection current was >0.7 A, and the highest WPE was obtained at 47.5% when the AlGaN thickness was 150 nm. Compared to the WPE of S1, where the peak WPE was 39.5%, the WPE of the optimized S2 structure can relatively increase by ~20%.

### 3.4. Device Performance of SiO_2_/ITO/p-AlGaN Cladding Structure (S3)

**Figure 8:**
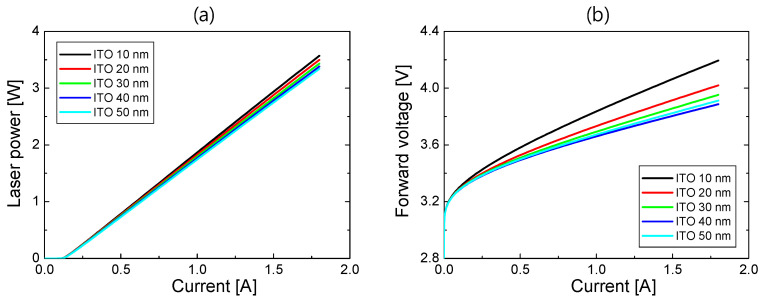
(**a**) *L*–*I* curves and (**b**) *V*–*I* curves of S3 for the ITO thicknesses from 10 to 50 nm when the p-AlGaN thickness is 75 nm.

Now, the LD device performance of S3 is described. Figure 8a,b respectively show the *L*–*I* and *V*–*I* curves for ITO thicknesses from 10 to 50 nm when the p-AlGaN thickness was 75 nm. In the *L*–*I* curves, the threshold current was ~0.12 A for all ITO thicknesses, almost the same as the threshold current of S2. The SE decreased from 2.12 to 1.99 W/A as the ITO thickness increased due to increased optical loss with the increasing ITO thickness. In the *V*–*I* curves, the forward voltage increased as the ITO thickness decreased. Since the current is injected laterally from Pd into the ITO and AlGaN/GaN layers, as shown in Figure 1c, the resistance of ITO could increase significantly for a thin ITO layer. However, the forward voltage increased slowly as the ITO thickness decreased from 50 to 20 nm, and it increased abruptly when the ITO thickness was further reduced to 10 nm.

**Figure 9:**
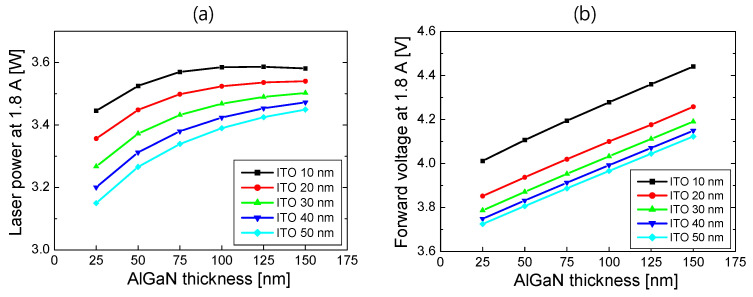
(**a**) Laser power at 1.8 A and (**b**) forward voltage at 1.8 A for the structure S3 are plotted as a function of the AlGaN thickness for ITO thicknesses from 10 to 50 nm.

In Figure 9, laser power and forward voltage at 1.8 A are plotted as a function of the AlGaN thickness for ITO thicknesses from 10 to 50 nm. As the thickness of ITO increased, the optical absorption in the ITO layer increased, and hence, the laser power decreased. The laser power also decreased as the AlGaN thickness decreased, which is attributed to the increased laser mode intensity in ITO with the decreasing AlGaN thickness. The laser power at 1.8 A for a 10 nm thick ITO was as high as 3.58 W, which is a little higher than that of 3.44 W at 1.8 A for S2 with the 300 nm thick ITO. Since no optical absorption is associated with the p-type electrodes in S3, higher SE and, thus, laser output power could be obtained. As shown in Figure 9b, the forward voltage increased as the AlGaN thickness or the ITO thickness decreased. The dependence of the forward voltage on the AlGaN thickness is the same as in the cases of S1 and S2. The increase in the forward voltage with the decreasing ITO thickness resulted from the increased resistance in the thin ITO layer, as mentioned previously.

**Figure 10:**
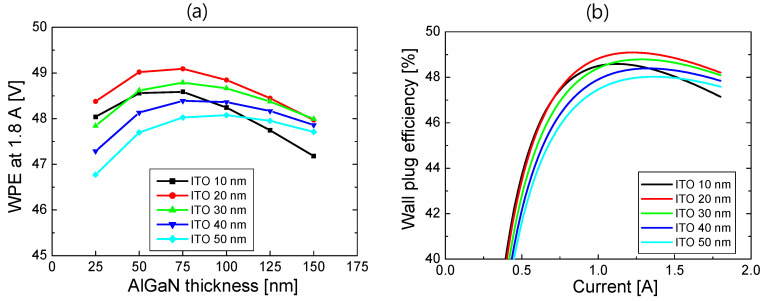
(**a**) WPE at 1.8 A for S3 is plotted as a function of the AlGaN thickness for the ITO thicknesses from 10 to 50 nm; (**b**) WPE of S3 as a function of current for the ITO thicknesses from 10 to 50 nm when the p-AlGaN thickness is 75 nm.

Using the simulation results of the *L*–*I* and *V*–*I* curves for S3, WPE is calculated as a function of current. In Figure 10a, the WPE at 1.8 A is plotted as a function of the AlGaN thickness for ITO thicknesses from 10 to 50 nm. The maximum WPE was obtained for the ITO thickness of 20 nm. As shown in Figure 10, both the laser power and forward voltage decreased with the increasing ITO thickness. At an ITO thickness of 20 nm, high laser power and low forward voltage were well-balanced, and hence, the maximum WPE could be obtained. The maximum WPE was obtained for the AlGaN thickness of ~75 nm for a given ITO thickness. This optimum value of the AlGaN thickness is half of the optimum AlGaN thickness of S2 and almost one-tenth of the optimum value of S1. Due to the remarkably reduced p-AlGaN thickness, it is possible to expect reduced operation voltage as well as improved laser output power compared to S2 and S3. Figure 10b shows WPE as a function of current for ITO thicknesses from 10 to 50 nm when the AlGaN thickness is 75 nm. The WPE exceeded 45% when the injection current was >0.6 A for all ITO thicknesses up to 50 nm. The maximum WPE was obtained at 49.1% when the ITO thickness was 20 nm. The WPE of the optimized S3 structure increased by 3.4% and 25% relative to the WPE of the optimized S2 and S1 structures, respectively.

**Figure 11:**
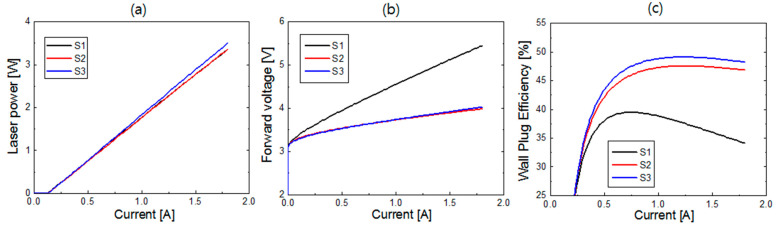
LD device performances for the optimized structures of S1, S2, and S3. (**a**) *L*–*I* curves; (**b**) *V*–*I* curves; (**c**) WPE as a function of current.

Figure 11 compares LD device performances between the optimized structures of S1, S2, and S3 for the highest WPE. The p-AlGaN thickness of the optimized S1, S2, and S3 was 700, 150, and 75 nm, respectively. The ITO thickness of the optimized S2 and S3 structures was 300 and 20 nm, respectively. The laser power of S1 and S2 is almost the same, and that of S3 is slightly higher than S1 and S2. This shows that the optical loss can be minimized in S3 by employing thin ITO and lossless SiO_2_ layers. In contrast, the forward voltage of S2 and S3 is significantly lower than S1, which is the result of the large decrease in the p-AlGaN thickness from S1 to S2 and S3. The forward voltage of S3 is slightly higher than S2. Although the p-AlGaN thickness of the optimized S3 structure is half the p-AlGaN thickness of the optimized S2 structure, the resistance in the thin ITO layer of S3 could increase due to lateral current injection from Pd.

As a result of the significant decrease in forward voltage, the WPE of S2 and S3 was greatly improved from S1. The WPE of S3 was a little higher than that of S2. The peak WPE values of S2 and S3 were 47.5% and 49.1%, respectively. That is, by employing the new LD structure with SiO_2_/ITO/p-AlGaN cladding layers, a small but noticeable increase in WPE can be expected compared to the optimized LD structure with ITO/p-AlGaN cladding layers. In addition, in S2 and S3, the high WPE of >45% could be maintained in a wide current range of >0.6 A. Furthermore, in S2 and especially in S3, thermal damage in the QW active region during the growth of p-AlGaN layers at a high temperature could be significantly alleviated because of the considerably shortened p-AlGaN cladding layer.

## 4. Conclusions

In this work, we performed numerical simulations on the device characteristics of InGaN blue LD structures with ITO and dielectric partial cladding layers. The optimum p-cladding structures to obtain the highest WPE were investigated as the thicknesses of ITO and p-AlGaN layers varied. For the optimum LD structure with ITO/p-AlGaN cladding layers, the p-AlGaN thickness was reduced to 150 nm, and the WPE increased by 20% relative to the WPE of conventional LD structures with a p-AlGaN cladding layer. Moreover, we proposed a new type of LD structure with SiO_2_/ITO/p-AlGaN cladding layers. For the optimum structure, the p-AlGaN thickness was further reduced to 75 nm, and the WPE increased relatively by 25% compared to the conventional LD structures. The reduced p-AlGaN thickness for the LD structures with ITO and SiO_2_ claddings decreased operation voltage significantly, which contributed to the remarkable increase in the WPE. The presented LD structures employing the ITO and SiO_2_ partial cladding layers are expected to be advantageous in achieving high-power GaN-based blue LDs with significantly improved efficiency.

## Data Availability

The data supporting the findings of this paper are available from the corresponding authors upon reasonable request.
